# A Qualitative Study of Impacts of the COVID-19 Pandemic on Lives in Adults with Attention Deficit Hyperactive Disorder in Japan

**DOI:** 10.3390/ijerph18042090

**Published:** 2021-02-21

**Authors:** Mizuho Ando, Toshinobu Takeda, Keiko Kumagai

**Affiliations:** 1 Center for Counseling and Development Support Services, University of Tsukuba, Bunkyo, Tokyo 1120012, Japan; 2 Department of Clinical Psychology, Ryukoku University, Fushimi, Kyoto 6128577, Japan; t-tak@umin.ac.jp; 3 Faculty of Human Sciences, University of Tsukuba, Bunkyo, Tokyo 1120012, Japan; kkumagai@human.tsukuba.ac.jp

**Keywords:** attention deficit hyperactive disorder, adult, COVID-19, qualitative study

## Abstract

(1) Background: COVID-19 may deteriorate some aspects among individuals with attention deficit hyperactivity disorder (ADHD). Although some positive aspects were reported during the pandemic, it remains unclear how COVID-19 qualitatively influences their living context; (2) Methods: this study employed interviews with four participants with ADHD during the declaration of emergency issued in Japan. The study was a part of ongoing coaching as a psychosocial intervention for ADHD, which was initiated long before the pandemic. The data were the answers to the question: “how are things going with participants during this pandemic?”. In a qualitative analysis, the researchers coded the data to identify different themes and sub-themes; (3) Results and Discussion: the qualitative data analysis yielded five themes: (1) Terrible feeling caused by frustration, stress, and anger; (2) Closeness due to the internal difficulties and conflict; (3) Deteriorating ADHD symptoms and executive function related matters; (4) Condition is the same as usual; and (5) Positive aspects associated with the self-lockdown. As a whole, these results show that the COVID-19 pandemic could be a factor in inducing psychological distress in the participants who adjust relatively better at work/school but did not do well at home before the pandemic; (4) Conclusions: this study indicates the need for special support for individuals with ADHD, especially those who originally had difficulties at home.

## 1. Introduction

In September 2020, more than a thousand people in Japan suffered from the COVID-19 disease due to the worldwide pandemic. According to the Ministry of Health, Labor and Welfare, as of 22 April 2020, the number of newly confirmed cases of COVID-19 was 420, and the cumulative number of confirmed cases of COVID-19, and the Coronavirus death tool were 11,919 and 287, respectively. Like other developed countries, although the number of patients was relatively low, the Japanese government issued a declaration of emergency on 7 April 2020. The uniqueness of this was that the declaration of emergency was not so restrictive. To prevent the spread of the virus, the Japanese people were “requested and encouraged” to maintain proper social distancing and to avoid the three Cs: “Closed spaces” with poor ventilation, “Crowded places” with many people nearby, and “Close-contact settings”, such as close-range conversations. With loose Japanese implementation for avoiding the occurrence of clusters, the reason for the low morbidity and mortality rate shown in Japan is still unknown, as is its impact on the people of Japan. This declaration of emergency had never been issued before in Japan, not even during the World War.

While the emergence of this pandemic has had a tremendous influence on individuals, it might be especially so for patients who have mental health problems, such as schizophrenia [1], as they are vulnerable and sensitive to environmental changes. However, the schizophrenic condition is not the only mental health problem, and individuals with developmental disabilities such as attention deficit hyperactive disorder (ADHD) may confront difficult situations due to the spread of COVID-19. ADHD is a neurodevelopmental disorder characterized by inattention, hyperactivity, and impulsivity [2]. These traits have been shown to impact various areas of people’s lives; in family, work, and/or school settings [3]. Consequently, this world-wide environmental change may worsen their ADHD symptoms and result in increasing their maladaptation to daily life. 

These types of concerns are additionally represented in the following statements. The Canadian ADHD Resource Alliance (CADDRA) (2000) stated that this worldwide emergency has increased isolation and difficulties in personal communication [4]. Similarly, the European ADHD Guidelines Group (EAGG) (2000) showed their concerns that behavioral problems are possibly increased among those with ADHD [5]. Specifically, increasing stress and anxiety, and difficulties with time management for parents who have children with ADHD [4]; as well as bedtime management becoming more difficult [5].

Losing environmental structure and daily routine can make individuals with ADHD more difficult [6], and they may lose opportunities to have education for special needs. These can be factors in the deterioration of their symptoms of ADHD [7]. Statistical analyses revealed that 54 percent of the children with ADHD showed decreasing concentration, 67 percent showed increased anger, and 56 percent showed deterioration of daily routine [7]. These findings indicate the possibility that individuals with ADHD could be a vulnerable group during the outbreak. Nonetheless, the results of this study additionally showed that more than half of the parents answered that there was no change or better in organization, being quiet, and listening to people. Moreover, McGrath (2020) stated that parents reported their child’s behavior and mood had improved during the school closure [8]. 

These findings indicate that, while staying home may have a negative impact on multiple factors, such as inattention, it may have positive aspects as well. However, the qualitative impact of COVID-19 on ADHD symptoms is unclear. Additionally, since the existing studies employed a retrospective design, uncertainty remains because individuals with ADHD were required to recall their experiences. Moreover, there are no studies on, in ongoing interactions, how individuals with ADHD experience their lives in detail when they engage in self-lockdown. Therefore, this study aims to qualitatively analyze the psychosocial and behavioral impact during Japan’s unique policy of self-lockdown, and reveal how environmental change affects the lives of individuals with ADHD symptoms.

## 2. Materials and Methods

### 2.1. Design

Based on an interpretive approach, this qualitative study was implemented as part of an ongoing coaching intervention for adults with ADHD; interviews were used to explore how these clients spent their time during self-lockdown. For this intervention, many of the cases received a combination of face-to-face and e-mail support; however, the coaching was changed to use a teleconference style instead of face-to-face contact. The first author conducted the interviews during the course of the coaching (Details please see Appendix A). 

The research team included researchers and practitioners in the area of neurodevelopmental disorders; they all had over twenty years of clinical experience at multiple settings such as hospitals and schools. There were some opportunities during practice for these researchers to listen to the participants experiences under the influence of COVID-19.

### 2.2. Participants

The participants were clients who had visited the counseling office from 2014 to 2020, and who regularly received support through an adaptive coaching intervention. They were selected using a convenience sampling method from the clients who had diagnoses of ADHD or suspected ADHD, and were receiving the intervention. The process proceeded through a teleconference between the first author and the individual participants. The study participants were over the age of twenty; the same ratio was obtained between males and females. To clarify the impact on ADHD in their daily lives, this study employed participants who were in school, work, and family settings. Since the interview process lasted for 15 min as a part of coaching intervention, there were no dropouts. 

Four Japanese clients (A, B, C, and D) participated, with their permission, in this study. No incentives or compensation were offered. The clients’ demographic data included age, sex, current employment, and residence. Their intellectual abilities were ascertained using the Wechsler adult intelligence scale (WAIS^TM^-III or IV) and ADHD symptoms by Conners’ adult ADHD rating scale (CAARS^TM^) (Table 1). A is a male, part-time worker, and in his 30s; he was diagnosed with ADHD combined type, and he resides with a parent. His full intellectual quotient (FIQ) is in the normal range, and his total ADHD index shows as above the threshold. B is female, in her 40s, a part-time worker diagnosed with ADHD combined type, who resides with her husband. Her FIQ is in the normal range and her ADHD index is the highest. C is in his 20s, a male university student diagnosed with suspected ADHD, who resides with his parents and siblings. His FIQ in 1 Standard Deviation (SD) is higher than the average, and his total ADHD index shows a moderate score. D is in her 30s, a female part-time worker diagnosed as having a neurodevelopmental disorder; she resides with her husband and children. Her FIQ in 1SD is higher than the average, and her total ADHD index is near 70.

### 2.3. Coaching

Coaching is a psychosocial intervention that supports by putting emphasis on the client’s autonomy and subjectivity. In coaching, a coach, as a supporter, helps clients to attain their goals by exchanging discourses that mainly consist of questions.

In this study, coaching was administered according to the method described in the papers on coaching [9,10,11]. Up until the emergency declaration call, sessions were conducted through face-to-face or online video meetings. Between sessions, e-mails were exchanged between the clients and the coach to mutually report and record the client’s situation. To avoid the 3Cs, from the declaration of the emergency to the end of this study, only online video meetings were used to carry out the sessions.

### 2.4. Ethical Issues

At the beginning of the session in the counseling room, informed consent was obtained from each client regarding protecting personal information and the possibility of using the session data for academic studies. Ethical review and approval were waived for this study, due to written consent being obtained at the Center for Counseling and Development Support Services, University of Tsukuba.

### 2.5. Data Collection

In this study, data was derived from conversations relating to the clients’ experience regarding situations during the declaration of emergency. More concretely, the data was the content of the answers when the clients were asked, “How are things going with you during this pandemic?” The intervener encouraged the clients to answer on their general impressions, and on the good and bad points of their lives during the pandemic. Conversations relevant to the study were derived from the first and second study-related sessions.

### 2.6. Data Analysis

Guided by the aims of the research, the first author developed a preliminary codebook that best represented the coachees’ reported experiences. Two other researchers experienced in qualitative studies independently coded two interviews using the preliminary codebook, and developed additional codes based on emergent themes using the qualitative data analysis software MAXQDA (Light Stone^®^, Tokyo, Japan). The coding from the two interviews was compared. Page-by-page comparisons were conducted, and the research team discussed the differences in the application or new code development until a consensus was reached. Modifications were made to the final codebook. One researcher used it to code all interviews. A sub-theme within each code was developed based on interview quotes. Finally, matrices were developed to explore responses across sites and compare relationships, repetition of themes within an interview and across interviews, patterns of responses across participants, and differences in responses.

## 3. Results

The results of the qualitative date analysis yielded five themes, as shown in Table 2. The themes were: (1) Terrible feeling caused by frustration, stress, and anger; (2) Closeness due to the internal difficulties and conflict; (3) Deteriorating ADHD symptoms and executive function-related matters; (4) Condition is the same as usual; and (5) Positive aspects associated with the self-lockdown. 

### 3.1. Terrible Feeling Caused by Frustration, Stress, and Anger

Clients described their experiences during COVID-19 with negative emotions, such as oppressive feelings due to being forced to stay home. This theme relates to time management, hyperactivity, and communication. The participants had a harder time with time management, especially the mother with ADHD.

*“I know that the attention span in children is short, but I was so frustrated that I lost my temper. My children tried to get my attention while I was working on my tasks, and my temper flared.”*

(Participant B)

*“I could not make a noise because my husband work at home. I love to quip a comedy on TV, using sewing machine, and so on. I need to be patient for so many things.”*

(Participant D)

While in self-lockdown, the participants who had hyperactivity could not release their energy and had difficulty in being quiet for the family members who worked at home. The clients were also sometimes frustrated by difficulties in communication with others. One participant stated that she lost her temper more easily than normal under the COVID-19 pandemic.

### 3.2. Closeness Due to the Internal Difficulties and Conflict

This theme includes the participants’ internal difficulties and conflict. They experienced boredom, hardship, concerns, and worries during self-lockdown.

*“Boring, unexciting, a sense of despair, and depressive feeling. I have been so frustrated, for long period of time, and it has been so hard that I feel this has never ended. I cannot go shopping, to the library, massage, and so on. I cannot do anything I want to. I do these things to avoid too much work.”*

(Participant D)

When this participant has hyperactivity, it is more difficult to deal with the stay-home situation. Under the normal situation, she goes out for social interactions with others to enjoy herself and to release her energy. 

### 3.3. Deteriorating ADHD Symptoms

Participants experience deteriorating inattention, hyperactivity, impulsivity, and executive dysfunction. This includes increasing difficulties in managing their behavior and controlling their motivation under the emergent situation.

*“It has been so long since I could manage fire in the kitchen, but I left to put a pan into the fire and I totally forgot about it. It might happen because I had something on my mind. I lost myself in thought if the grocery store is open, then if it opens as usual, I should wear the plastic gloves, and so on.”*

(Participant D)

*“It is unable to manage time of my family members at home while I am not good at time management.”*

(Participant B)

As with this participant, deteriorating ADHD symptoms are described in various ways. They experienced making careless mistakes due to the situation being different from the normal. Moreover, participants described it being difficult to manage their schedule, because people frequently ask to change the schedule and sometimes they do so the day before the events. 

### 3.4. The Condition Is the Same as Usual

For this theme, the participants described their experiences as the same as usual: There was nothing in particular that had changed in a negative or a positive sense.

*“I feel good about the cadences of my daily life.”*

(Participant C)

This participant described their situation under self-lockdown as the same as normal in both family and work. There were no influences due to self-lockdown and the spread of COVID-19. Some stated that remote work had already started at a certain setting, and therefore they were not affected by a change in working style.

### 3.5. Positive Aspects Associated with the Self-Lockdown

Some participants mentioned the positive aspects of the self-lockdown. This included how their lives turned out to be easier and more comfortable than normal in some aspects, such as their daily routine, sleep duration, and task management.

*“Surprisingly, I can lead a regulated life!”*

(Participant A)

*“I am a repeater mainly due to the failure turning in my papers, but since staying home, I have been able to hand them in for 100%, which is unbelievable. I can proceed my assignment because the professors send the title and contents of the assignment clearly on the portal, so I do not miss them. It is so nice to work at my own pace.”*

(Participant C)

These participants stated that they could keep their own pace because they did not need to pay attention to others to keep from missing anything at work. In addition, since they did not need to pay attention to others, it was very easy for them to escape from the social orders and rules in Japan. They did not need to get prepared for work and school, which usually requires their energy not to be late, and to bring everything that they need for work or school. Moreover, since they are surrounded with their favorite things in their own room, many things inspire them with new ideas, and they have become more creative with work.

## 4. Discussion

This qualitative study was conducted to elucidate the situation of adults with ADHD during the self-restraint period in response to the COVID-19 epidemic. The patients were asked how they were doing during this period, and their narratives were analyzed qualitatively. Although some were shown to have had a more difficult time due to a deterioration in their executive functions, others had an easier time in comparison. Previous studies revealed that some symptoms of ADHD became worse, while others did not, and some even improved [7]. This study confirmed these findings, that there are both positive and negative aspects in reactions regarding the pandemic. However, since these studies did not offer detailed information on how the patients experienced this unusual period, this current study aimed to illuminate the qualitative aspects of the patients’ experience.

The first theme, “Terrible feeling caused by frustration, stress, and anger,” pertains to the negative emotions during the self-restraint period. Although most people were experiencing these negative emotions, more or less, including frustration and anger, during the period, our study showed that the negative emotions were exacerbated by difficulties in time management and confinement in the home in adults with ADHD. These negative emotions, such as irritation, have a subsequent negative affect on their communication.

Temporal disorganization is one of the main difficulties of executive functioning in ADHD. While every family member stays home during the pandemic, parents with ADHD have to manage their own time as well as that of their children. McGrath (2020) and Zhang (2020) reported that parents of ADHD children favorably rated their children’s behavioral problems during the temporary closure of the schools [7,8]. However, a study on how the behaviors of parents with ADHD changed during this difficult time is not yet available. One of the cases in this study was a parent with ADHD, and she got overwhelmed in rearing her child during the self-restraint period. Existing papers on mothers with ADHD found that there is a negative correlation between the strength of the ADHD symptoms in mothers and their subjective confidence in controlling their children’s behavior [12]. Other studies have found that the mothers’ ADHD had a relationship with overreacting against their children, difficulty in controlling their children’s behavior, and low satisfaction with rearing children [13,14]. These findings are the characteristics of behaviors in parents with ADHD during ordinary times. However, during the self-restraint period, in cases where parents have to stay with their children 24/7, the burden of parents with ADHD in rearing children gets significantly heavier. Most parents, including parents with ADHD, find it difficult to schedule their children’s activities.

Due to their own deficit in time management skills, parents with ADHD have a much more difficult time in managing their children’s activities [15]. In general, while staying home was encouraged during the self-restraint period, the family system, including parents with ADHD, could potentially exhibit a vicious cycle; therefore, early intervention and how to intervene in this situation should be considered in the future.

The theme “Deteriorating ADHD symptoms and executive function-related matters” includes problems in managing time and money, exacerbating inattention, and maintenance of one’s routine. The sudden onset of unfamiliar daily routines made adults with ADHD think about many issues simultaneously; consequently, both their inattention and their distractibility became worse, as Zhang (2020) has already pointed out [7]. 

Financial management became less efficient during the self-restrained period. This condition could be related to abuse/dependency. Compulsive buying, which is not yet included in DSM or ICD diagnoses, is a psychosocial problem, and it should be confronted as an important mental health issue. Compulsive buying is frequently comorbid with anxiety disorders and eating disorders and, as some have pointed out, has some commonality with impulsivity [16,17]. In adults with ADHD, their impulsivity, combined with the large amount of time in the home and the lack of physical activities during the pandemic, makes them susceptible to compulsive buying, which could lead to deterioration in their lives. Psychosocial support, such as stress management and psychoeducation, should be provided to this vulnerable population to help them keep track of their finances. 

The unclear future could have posed ADHD patients difficulties in making a new daily routine according to the new situation. Originally, it was often pointed out that individuals with ADHD have difficulty in making and keeping a daily routine [2]. To compensate for this difficulty, some ADHD patients may use a daily schedule or a checklist. However, during the pandemic, the daily schedule may change day by day (e.g., telework or office work) and, according to the change, their schedule could be altered frequently. In this situation, it is easily understood that their compensation strategy will not completely work; they forget their appointments (e.g., Zoom meetings) and make careless mistakes.

While some reported a more difficult time during the self-restraint, it cannot be overlooked that there were also themes including “Condition is the same as usual” and “Positive aspects associated with the self-lockdown.” The remarks that individuals with developmental disorders are vulnerable to unusual situations such as the COVID-19 pandemic is important; however, it could be equally important to shed light on the positive side of this pandemic, which they enjoy. According to a recent report, compared to adults with typical development, who had no change in daily difficulties in their lives, adults with high-functioning autism marked a drastic decline in daily difficulties in their lives [18]. It is highly likely that adults with developmental disorders, such as ASD and ADHD, experience a very difficult time on a routine basis in many aspects of their work lives, such as in communicating, flexibility, time management, organization, and planning, and that the heavy load on their lives has lifted during the self-restraint period. 

In this study, one patient reported that he could hand in papers during the pandemic better than during ordinary times. Due to executive disfunction, many people with ADHD struggle with their homework assignments because it requires skills to manage many steps to complete their assignment at home. Langberg et al. (2020) showed nine steps for a homework assignment [19]. However, while staying home due to the pandemic, processes 1, student records assignments accurately and in sufficient detail; 2, student ensures materials needed for homework are brought home; 4, student manages time after school effectively; and 6, student ensures materials and assignments are brought back to school are not necessarily required in an online class. For example, strict time management regarding process 4 is not needed, as you can use your time freely during the pandemic. The situation when 4 out of 7 processes are not required in an online class highlights the difficult steps in handing in assignments for a patient who frequently fails in submitting assignments during ordinary times. 

While some have difficulty in keeping routines in this extraordinary situation, others reported improvement in their daily rhythm, which reveals their poor time management skill during ordinary times. During the self-restraint period, time and effort is saved both from commuting to the workplace and from the morning routines, such as changing clothes. This has three major merits for ADHD patients. First, they have more free time than usual. Some adults with ADHD could take advantage of a few hours of bonus time during the pandemic. In particular, since ADHD is often comorbid with sleep problems [20], this extra free time would work for them. Second, adults with ADHD spent more time in getting themselves ready for work (e.g., dressing themselves, grooming, checking their belongings) [21]. Third, after leaving home, they usually get exhausted in making it for the right train, checking their documents for work, and being careful not to miss their stop when they reach the workplace; however, they have more energy to do their work without the time-consuming and exhausting commute. Although the long-term effect of this remains to be seen, their narratives teach us how these daily hassles drain the life out of them. 

**Figure 1:**
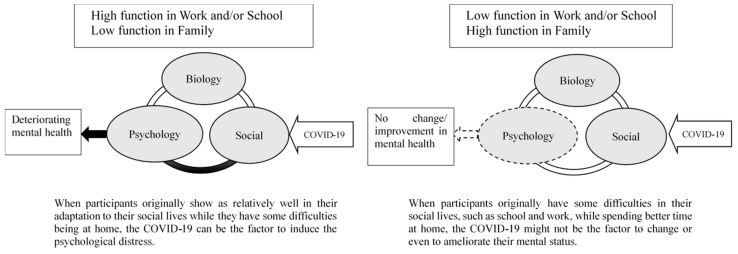
Psychosocial effect on ADHD during the outbreak.

In this study, for a female participant with ADHD who originally had difficulties in her home with taking care of the home and children, working outside would provide her with a shelter from hardship in the home. However, during the pandemic, she was not allowed to go outside without essential reasons during the self-lockdown. As a result, staying home for a long time would deteriorate her mental health. In Japanese society, where women mainly take care of the home and children, women with ADHD would be easily overwhelmed by their dual task (i.e., online work and daily hassles in home) during the pandemic. In this respect, a considerable portion of women with ADHD would need some kind of support during emergency situations. In comparison, for individuals who are originally doing well at their home but have struggled to deal with something outside, staying home can have a minimum negative impact or even positive impact on their lives (See Figure 1). 

This study has limitations in terms of credibility and reliability. It analyzed narratives of clients with whom there was an established coaching rapport. Therefore, without any hesitation in speaking frankly, their narratives may reflect their actual situation during the self-restraint. However, given the possibility that their narratives mainly reflect what happened just before the interview, there is no guarantee that their narratives perfectly reveal their whole experiences during the pandemic. The benefit of a qualitative study is to pick up individuals’ detailed experiences, which a quantitative study may overlook. Although individual experiences cannot be easily generalized, personal experiences must be analyzed in both a quantitative and a qualitative manner to better understand the experiences more deeply and utilize them to help or support clients.

## 5. Conclusions

The influences of the self-restraint situation on adults with ADHD were qualitatively analyzed. They varied among the clients. While the situation negatively affected some clients’ lives, it positively affected or had little impact on the lives of others. The positive experiences of adults with ADHD during the declaration of emergency elucidated the difficult times that they were experiencing during ordinary times. Some patients showed a negative change in their emotions and behavior due to worsened ADHD symptoms during the pandemic. Tailor-made support or intervention should be implemented considering individual circumstances and characteristics, including the heterogeneity of ADHD symptoms.

## Figures and Tables

**Table 1:** The demographic and clinical data of the participants.

	Age	Sex	Employment	Household Composition	FIQ	CAARS ADHD Index	ADHD Type
A	30s	Male	Part-time worker	Parent	Average	Markedly atypical	Combined
B	40s	Female	Part-time worker	Partner	Average	Markedly atypical	Combined
C	20s	Male	Student	Parents; siblings	High average	Moderately atypical	Inattention
D	30s	Female	Part-time worker	Partner; children	High average	Moderately atypical	Combined

**Table 2:** Theme and Sub-theme.

Theme	Sub-Theme	Participants
Terrible feeling caused by frustration, stress and anger		
	Time management	B
	Overcoming hyperactivity	D
	Communication	D
Closeness due to the internal difficulties and conflict		
	Hardship	B
	Guilt	B
	Confinements	A
	Digression	A
	Boredom	D
	Agony	D
Deteriorating ADHD symptoms and executive function related matter		
	Time-management	B
	Procrastination	B
	Money-management	A
	Scheduling	D
	Inattention	D
	Routine	D
Condition is the same as usual		
	Normal	C
	No influence	D
Positive aspects associated with the self-lockdown		
	My own pace	B
	Effortless time management	B
	Successful assignment	C
	Disciplined life	A
	Less fatigue	A
	Free from rules-based order	A
	Imagination	A

## Data Availability

No new data were created or analyzed in this study. Data sharing is not applicable to this article.

## Appendix A. Details of the Interviews

Settings	The first author implemented the interviews with clients using teleconferencing.
Number of the interviews	Once per participant.
Duration of the interviews	Interview took around 15 min.
Timing of the interviews	The timing of the interview was at the beginning of the coaching intervention.
Participants recruitments	All participants were the clients who get ADHD coaching from the first author.
How to record the interviews	During the interviews, the first author who implemented the interviews took a careful note of the conversation.
Interview schedule	All data provided by the interviews was collected during the state of emergency in 2020.
